# Progress of Cholera

**Published:** 1849-02

**Authors:** 


*Progress of Cholera.*—Dr. Taylor, in a report from the Committee of Public Health, stated, that the total number of cases admitted into the Cholera Hospital of Edinburgh, from the 30th of October, the date of the epidemic was 177, of whom 106 died, 57 had been cured, 2 had been sent to the Infirmary as not being cholera cases, and 12 were still under treatment. With respect to the habits of the persons admitted, he might state, that out of 127 of them, there were 42 males and 85 females, of whom 15 males and 15 females were of dissipated habits; 3 males and 22 females were of irregular habits; 2 males and 14 females were doubtful in their habits; and 22 males and 34 females were of sober habits; but out of the latter class had to be deducted 12 boys and 15 girls under fourteen years of age, which, therefore, left a very small proportion of sober people. Still later accounts show an increase both in the number of cases and of the mortality.

The total number of cases at Leith have been 298; deaths 115, recoveries 175, remaining 8.—*Med. News.*

